# The Editor's Letter-Box

**Published:** 1914-05-23

**Authors:** Arthur J. Brock

**Affiliations:** Edinburgh


*To the Editor of* The Hospital.

Sir,—Dr. Johnston asked whether it is not faith in himself that keeps a man off the panel? Well, obviously enough, it is, in many cases. I was merely concerned to point out that such excellent motives were not the only, or indeed the main, ones which kept many other men off the panel. For example, the possession of a fat banking account or an income derived from non-professional sources would sufficiently explain many cases of abstention.

Ordinary medical and surgical degrees are little enough indication, in all conscience, of a man's professional capacities. The proposed "Medical Directory" degree of *Non-Pan.*, however, would be of even less value. It might mean anything or nothing. It would include not only the man who had stayed off the panel on principle, but also the sixpenny doctor from the slums, and Sir Diddelem MacVaccine from the West End, who battens on overfed, plethoric duchesses and society *demi-mondaines* of both sexes. No, no, Sir, the degree of Non-Pan. (*Lond. et Edin.*) would in itself afford the long-suffering public no criterion of professional aptitude or virtue.

One other point, it may be taken as an established rule that the man who has most faith in himself and his art is the last to wish his merits advertised in directories. I hasten, of course, to add my conviction that Dr. Thomas Johnston is a shining example of this rule.—I am, yours faithfully,

Arthur J. Brock, M.D.

Edinburgh, May 16. 1914.

